# Overexpression of the microRNA miR-433 promotes resistance to paclitaxel through the induction of cellular senescence in ovarian cancer cells

**DOI:** 10.1002/cam4.409

**Published:** 2015-02-15

**Authors:** Karolina Weiner-Gorzel, Eugene Dempsey, Malgorzata Milewska, Aloysius McGoldrick, Valerie Toh, Aoibheann Walsh, Sinead Lindsay, Luke Gubbins, Aoife Cannon, Daniel Sharpe, Jacintha O'Sullivan, Madeline Murphy, Stephen F Madden, Malcolm Kell, Amanda McCann, Fiona Furlong

**Affiliations:** 1 UCD School of Medicine and Medical Science (SMMS), UCD Conway Institute of Biomolecular and Biomedical Research, University College DublinDublin 4, Ireland; 2 UCD School of Biomolecular and Biomedical Science, Conway Institute, University College DublinDublin 4, Ireland; 3 Systems Biology Ireland, University College DublinDublin 4, Ireland; 4 Molecular Department of Surgery, Institute of Molecular Medicine, Trinity Centre for Health Sciences, St James's HospitalDublin 8, Ireland; 5 School of Pharmacy, Queen's University of BelfastBelfast, Northern Ireland, United Kingdom; 6 Molecular Therapeutics for Cancer Ireland, National Institute for Cellular Biotechnology, Dublin City UniversityGlasnevin, Dublin 9, Ireland; 7 Department of Surgery, Mater Misericordiae University HospitalDublin 7, Ireland

**Keywords:** CDK6, chemoresistance, miR-433, ovarian cancer, senescence

## Abstract

Annually, ovarian cancer (OC) affects 240,000 women worldwide and is the most lethal gynecological malignancy. High-grade serous OC (HGSOC) is the most common and aggressive OC subtype, characterized by widespread genome changes and chromosomal instability and is consequently poorly responsive to chemotherapy treatment. The objective of this study was to investigate the role of the microRNA miR-433 in the cellular response of OC cells to paclitaxel treatment. We show that stable miR-433 expression in A2780 OC cells results in the induction of cellular senescence demonstrated by morphological changes, downregulation of phosphorylated retinoblastoma (p-Rb), and an increase in *β*-galactosidase activity. Furthermore, in silico analysis identified four possible miR-433 target genes associated with cellular senescence: cyclin-dependent kinase 6 (CDK6), MAPK14, E2F3, and CDKN2A. Mechanistically, we demonstrate that downregulation of p-Rb is attributable to a *miR-433-dependent* downregulation of CDK6, establishing it as a novel miR-433 associated gene. Interestingly, we show that high miR-433 expressing cells release miR-433 into the growth media via exosomes which in turn can induce a senescence bystander effect. Furthermore, in relation to a chemotherapeutic response, quantitative real-time polymerase chain reaction (qRT-PCR) analysis revealed that only PEO1 and PEO4 OC cells with the highest miR-433 expression survive paclitaxel treatment. Our data highlight how the aberrant expression of miR-433 can adversely affect intracellular signaling to mediate chemoresistance in OC cells by driving cellular senescence.

## Introduction

Worldwide ovarian cancer affects over 240,000 women annually. The most common subtype among ovarian cancer malignancies is high-grade serous ovarian cancer (HGSOC) which accounts for ∼70% of all ovarian cancer presentations 1. On average, the 5-year survival rate for HGSOC ranges from 20% to 40% and is primarily dependent on the initial stage of diagnosis 2. The standard management of ovarian cancer includes cytoreductive surgery followed by adjuvant chemotherapy consisting of a DNA-binding platinum agent (carboplatin) and a microtubule-stabilizing agent (paclitaxel). Despite the often initial favorable response, patients unfortunately usually relapse within 6–12 months 3. The rational given for such a high recurrence rate is predominantly associated with the “repopulation hypothesis” which assumes that recurrence depends on the ability of cancer cells to survive chemotherapy due to an intrinsic or acquired resistance 2. Resistance to the microtubule-stabilizing agent, paclitaxel, can be associated with multiple mechanisms including an increased efflux of the drug, aberrant paclitaxel binding 4 or a disruption of the spindle assembly checkpoint (SAC) function 5. Importantly, molecular pathways targeted by paclitaxel and carboplatin chemotherapies, determine their best efficacy toward actively dividing cells 4. Thus, cell cycle arrest and subsequent cellular apoptosis is the predominant mechanism of action of these chemotherapies.

Cellular senescence is a state of cell cycle arrest initiated by various stimuli such as telomere shortening, stress factors (oncogenes), or chemical moieties (e.g., chemotherapies) 6. Due to the growth repression feature, cellular senescence is widely regarded as a tumor suppressor mechanism 7. This view, however, has recently been challenged, whereby it is widely believed that cellular senescence induction can contribute to the development and further progression of cancer cells 8. Senescent cells demonstrate various characteristic features including changes in morphology and molecular signaling. The initiation and maintenance of senescence is mediated by the p53/p21 and p16/Retinoblastoma (Rb) pathways 8. An increase in the p16 and p21 cyclin-dependent kinase inhibitors negatively regulates Cyclin/cyclin-dependent kinase (CDK) complexes and prevents the phosphorylation of Rb 9. Therefore, hypophosphorylated Rb is a hallmark of cell cycle arrest. Dysregulation in these two molecular pathways is a feature of many cancers including ovarian tumors 10.

For the last decade, extensive research has identified a pivotal role of microRNAs in cancer 11. We have previously published that high expression of miR-433 is significantly associated with poor progression-free survival (PFS) in patients with HGSOC and demonstrated that downregulation of the mitotic arrest deficiency protein MAD2 (also known as MAD2L1) by miR-433 induced cellular chemoresistance to paclitaxel 5. Shih et al., 2011 also demonstrated an association between miR-433 and poorer survival in ovarian cancer, while others have demonstrated altered miR-433 expression in gastric cancer 12, myeloproliferative neoplasms 13 and lung dysplasia 14. Additionally, many of the published miR-433 protein targets are strongly associated with cancer including GRB2, SFRP2, CREB1, and HDAC6 15–19. Moreover, miR-433 expression is correlated with the inhibition of cell migration, proliferation, and differentiation 13,17,20. To date, however, the mechanism by which miR-433 expression promotes resistance to paclitaxel treatment in cancer cells is not known.

Here, we examined the underlying mechanism of miR-433-mediated chemoresistance to the chemotherapeutic paclitaxel in ovarian cancer. We demonstrate that increased resistance to paclitaxel in miR-433 overexpressing ovarian cancer cell lines is not only mediated through the inhibition of apoptosis 5 but also through miR-433-induced cellular senescence. Moreover, we present novel data showing the downregulation of CDK6 by miR-433 as a putative mediator of miR-433-induced senescence. Additionally, we demonstrate that growth-conditioned media (GCM) from miR-433 expressing cells have the potential to modulate the tumor microenvironment by inducing growth inhibition and cellular senescence in neighboring cells. Our data highlight how the aberrant expression of miR-433 and the miR-433 protein targets can adversely affect intracellular signaling activities to mediate chemoresistance in ovarian cancer cells.

## Materials and Methods

### Tissue culture

A2780, PEO1, and PEO4 cells were obtained from the European Collection of Cell Cultures (ECACC) and cultured in RPMI (Roswell Park Memorial Institute medium). However this name is not used in the scientific literature. 1640 medium supplemented with 10% v/v FCS (fetal calf serum) and 0.3 mg/mL glutamine. The miR-433 stable cell lines were cultured in RPMI 1640 medium supplemented with 10% v/v FCS, 0.3 mg/mL glutamine, and puromycin (4 *μ*g/mL) as a selection antibiotic. All cells were maintained at 37°C with 5% CO_2_ and were routinely tested and proven negative for mycoplasma contamination.

### Generation of the A2780 ovarian cancer cell line with ectopic expression of hsa-miR-433

A2780 cells stably expressing hsa-miR-433 were constructed using commercially available plasmids (shMIMIC Lentiviral Human microRNA nonsilencing control and miRIDIAN microRNA hsa-miR-433 shMIMIC), both harboring a TurboGFP reporter (HMR5872 and VSH5841-202526908, respectively, Thermo Fisher Scientific Inc., Waltham, MA, USA) and used according to the manufacturer's protocol. Stable selection of transfectants was ensured by a puromycin (4 *μ*g/mL) selection process to generate a miR-433 or miR-control expressing cell lines. Successful transduction was assessed by positive tGFP expression, microRNA expression, and target gene downregulation.

### SDS-PAGE and Western blotting

Sodiumdodecyl sulphate polyacrylamide gel electrophoresis (SDS-PAGE) and Western blotting were performed as described previously 5. The following antibodies were used: MAD2 (BD BioScience, San Jose, CA, USA), HDAC6 (Millipore, Billerica, MA, USA), Caspase 3, p21, poly (ADP-ribose) polymerase (PARP), CDK6 and p-Rb (Ser 807/811) (Cell Signalling, Danvers, MA, USA), *β*-actin and p16 (Santa Cruz Biotechnology, Dallas, Texas, USA), beta (*β*)-galactosidase (Abcam, Cambridge, UK).

### Cell viability assay

Ovarian cancer cells stably expressing miRIDIAN hsa-miR-433 or control shMIMIC were treated with (10, 25, 50 nmol/L) paclitaxel (Sigma, St. Louis, MO, USA) for 24 h and analyzed by the MTT assay, as previously described.

### Colony formation assay

Stable miR-control and miR-433 expressing cells were seeded onto six-well plates at a density of 1500 cells per well as previously described 21. Resultant colonies were fixed with 4% paraphormaldehyde (Electron Microscopy Science, Hatfield, PA, USA), stained with crystal violet (10% v/v—Sigma) then counted and the % colony formation was calculated using control untreated cells as a calibrator.

### *β*-galactosidase staining

Stable miR-control and miR-433 A2780 expressing cells were seeded onto six-well plates at a density of 3 × 10^5^ cells per well and allowed to grow for 48 h. The cells were then fixed and stained using a senescence *β*-galactosidase staining kit (Cell Signalling) according to the manufacturer's instructions.

### Quantitative real-time PCR

For the analysis of miR-433 expression in miR-433 stable cell lines, total RNA was isolated from the cell pellets using the TRIzol® Reagent (Invitrogen, Life Technologies, Grand Island, NY, USA) based on the manufacturer's protocol. RNA (50 ng) was reverse transcribed using the TaqMan® MicroRNA reverse transcription kit (Applied Biosystems). Quantitative real-time polymerase chain reaction (qRT-PCR) was performed using TaqMan® chemistry (Applied Biosystems) on 9700 Thermocycler (Applied Biosystems) to analyze the expression of each microRNA by the comparative delta Ct method normalized using RNU6B as endogenous control.

For the analysis of miR-433 expression in ovarian cancer cell lines, total RNA was isolated from cultured cells using the mirVana™ miRNA Isolation Kit (Ambion, Grand Island, NY, USA) according to the manufacturer's instructions. RNA (1 *μ*g) was reverse transcribed using the miScript II RT Kit (Qiagen). qRT-PCR was performed using SYBR-based chemistry (Qiagen, Germantown, MD, USA) on the Lightcycler 480 system (Roche, Clare, Ireland) to analyze the expression of each microRNA by the comparative delta Ct method normalized using RNU6B as endogenous control.

### Transient transfection

Cells were transiently transfected with 100 nmol/L of pre-miR-433, scrambled control miRNA, anti-miR-433, or anti-miR-control (Dharmacon, Lafayette, CO, USA) using TransIT-X2® (Mirrus, Madison, WI, USA) transfecting reagent according to the manufacturer's protocol.

### Coculture with growth condition media

GCM was obtained from ∼80% confluent A2780, PEO1, and PEO4 cells or miR-control-stable and miR-433-stable A2780 cells. Next, GCM was centrifuged at 125×g for 5 min and filtered to remove debris and dead cells and added to recipient A2780 or miR-433-stable cells, respectively. This procedure replacing fresh GCM was repeated every 24 h for 96 h. Following this treatment, Western blot analysis or MTT viability assay was performed.

### MicroRNA target prediction

Target genes of individual miRNAs were calculated using seven different databases: DIANA-microT 22, miRDB 23 and TargetScan 24, miRANDA, PITA 25, RNA22, and miRWalk 26. The results for DIANA-microT, miRDB, and TargetScan were updated to include the latest versions of these databases. Genes were assigned a value of 1 per algorithm where a positive prediction was made. These values were summed and only genes with a score of 5 or higher (positive prediction by five of seven databases) were included in further analysis.

Protein–Protein Interaction (PPI) networks were generated within Cytoscape (version 3.01) 27. All interaction data were obtained from STRING ver 9.05 using the data import function through CluePedia ver 1.07. Only experimentally validated and curated interactions were used to build PPI networks. Gene Ontology (GO) function enrichment for downstream target genes was performed in Cytoscape using the (ClueGo app (version 2.07), Cordeliers Research Center, Paris, France) 28.

### Statistical analysis

All data are presented as the mean ± SEM for at least three independent experiments. For each experiment, the statistical tests are indicated in the results section. Student's *t*-test was conducted using Prism 5 (Graphpad Software, La Jolla, CA, USA) to compare the means.

## Results

### Stable expression of miR-433 in A2780 cells attenuates cellular apoptosis and results in a distinct morphological change

We previously demonstrated that transient overexpression of pre-miR-433 in A2780 cells decreased the apoptotic response of ovarian cancer cells to paclitaxel treatment 5. Here, we have investigated the long-term effects of miR-433 overexpression in ovarian cancer cells. To address this, a miR-433 overexpressing A2780 cell line and a matched miR-control (miR-433 negative) were generated. To ensure that only transduced cells were further cultured, puromycin selection was applied to the stable transfectants. Cells were also routinely monitored for the retention of GFP as a positive control of miR-433 or miR-control expression.

**Figure 1:**
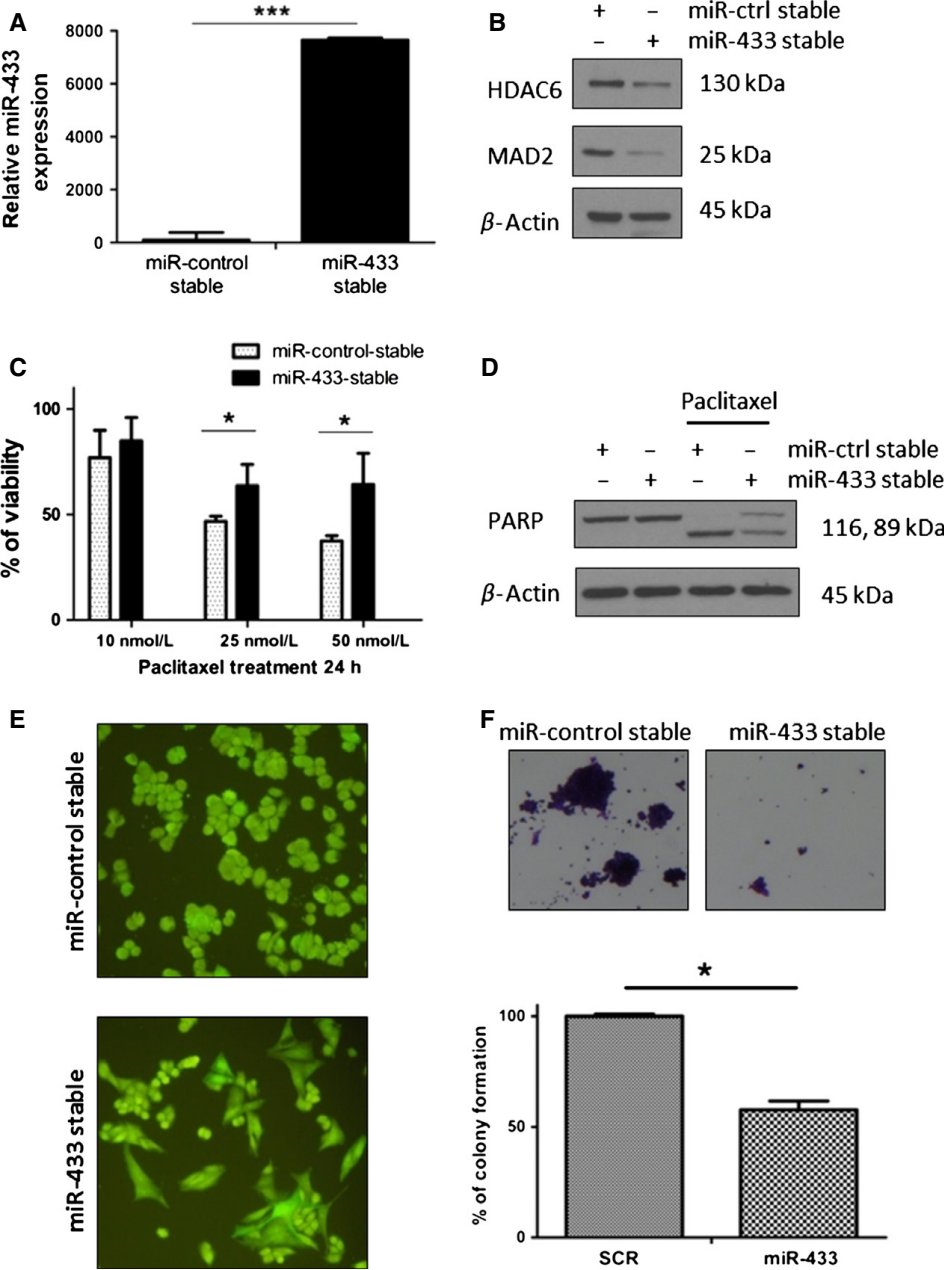
Stable expression of miR-433 in A2780 cells attenuates cellular apoptosis and results in a distinct morphological and proliferative change. (A) Quantitative real-time PCR (qRT-PCR) values for miR-433 in the miR-433-stable A2780 cells compared to miR-433-stable cells showing a significant (*P* < 0.05) fold increase in miR-433 levels. (B) Western blot analysis showing downregulation of two miR-433 targets, MAD2 and HDAC6 in the A2780 miR-433-stable expressing cell line. (C) Histogram representation of the response of the stable miR-433 versus control-miR cell lines post 24 h paclitaxel (10, 25, 50 nmol/L) treatment. Student's *t*-test was used for the comparison of means and demonstrates an increased resistance of the miR-433-stable cell line compared to controls at 25 and 50 nmol/L paclitaxel (*P* < 0.05). (D) Western blot analysis of poly (ADP-ribose) polymerase (PARP) cleavage in the miR-433-stable and miR-control-stable cell line treated with 50 nmol/L paclitaxel for 24 h demonstrating a decreased PARP cleavage in the miR-433-stable expressing A2780 cells. (E) Increased miR-433 expression influences the cellular morphology of A2780 cells appreciated by green fluorescent protein (GFP) expression with a resultant flattened and enlarged cellular morphology. Magnification 20×. (F) Colony forming assay of miR-control and miR-433 stable tranfected A2780 cells stained with crystal violet (magnification 4×) demonstrating a significantly lower colony formation ability in the miR-433 stable transfectants. Error bars represent SEM. *P < 0.05, ***P < 0.001.

miR-433 expression was confirmed by qRT-PCR analysis and was significantly upregulated in the stably transfected cells compared to control (*P* < 0.001) (Fig.1A). To confirm that stable overexpressed miR-433 was functionally active, we examined the protein expression of two known miR-433 target genes, MAD2 5 and HDAC6 15. The levels of both proteins were downregulated in the miR-433 overexpressing cells when compared to controls (Fig.1B).

Previously, we have reported that transient transfection of miR-433 rendered A2780 cells more resistant to paclitaxel treatment 5. Here, we investigated if stable miR-433 expression would also enhance paclitaxel resistance. Transduced A2780 cells were subjected to a range of paclitaxel concentrations. At 25 and 50 nmol/L, miR-433 expressing cells demonstrated greater cell viability compared to controls (*P* < 0.05) (Fig.1C). Furthermore, Western blot analysis demonstrated diminished PARP cleavage in the stable miR-433 expressing cells, suggesting a miR-433-dependent reduction in apoptosis (Fig.1D). Therefore, a similar apoptotic response is obtained irrespective of whether miR-433 is expressed transiently 5 or stably.

Following 1 week of functional miR-control/miR-433 expression evidenced by GFP fluorescence, we observed a significant morphological difference between the miR-433-stable and the miR-control-stable cells (Fig.1E). Specifically, miR-433-stable cells appeared more flattened, enlarged and enriched with cytoplasmic granules compared to control cells (Fig.1E). Additionally, miR-433 stable cells formed significantly less colonies compared to miR-controls (Fig.1F). Taken together these results demonstrate that miR-433 induced both phenotypic and morphological changes in cancer cells. Importantly, the morphological changes were not apparent in cells transiently transfected with miR-433 5.

### Bioinformatic identification of putative miR-433 target genes identifies potential links to cellular senescence

The resistance to paclitaxel and changes in growth and morphological characteristics suggested that over expression of miR-433 was inducing cellular senescence in the stable transfected A2780 cells. To explore this hypothesis further, we first set out to identify additional putative targets of miR-433 using a bioinformatic approach. To identify candidate miR-433 targets, we utilized seven different prediction algorithms (TargetScan, DIANA Micro-T, miRWalk, RNA22, miRANDA, PITA, and miRDb). From this analysis, we identified possible 6224 candidate genes. However, applying the principle that a candidate gene must be detected by five out of seven algorithms, this list was reduced to 1204 genes (Table S1). In this analysis, both MAD2 (also known as MAD2L1) and HDAC6 were identified as targets by seven and five algorithms respectively, thus, giving credibility in the candidate gene selection criteria.

**Figure 2:**
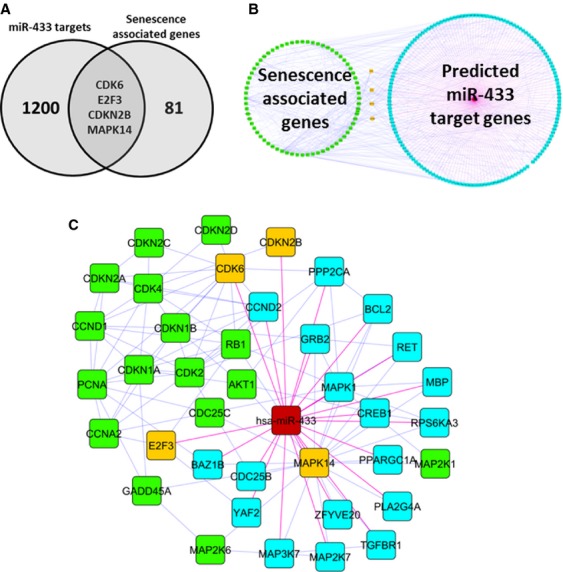
Bioinformatic analysis highlights miR-433 potential influence on cellular senescence. (A) Venn diagram showing overlapping genes (CDK6, MAPK14, CDKN2B, and E2F3) between senescence-associated genes and miR-433 target genes. (B) Overview of the interaction between miR-433 target genes (blue nodes) and senescence-associated genes (blue nodes), overlapping nodes are colored yellow. (C) Focus on overlapping genes (yellow nodes) and first degree neighbors in both the senescence-associated genes (green nodes) or predicted miR-433 targets (blue nodes). Blue edges indicate experimentally validated protein–protein interactions, magenta edges indicate predicted interactions with miR-433 (red node).

Having identified candidate miR-433 target genes, we next wanted to determine the potential for these genes to impact on cellular senescence. Well-established genes associated with cellular senescence were identified from the literature, yielding a list of approximately 86 genes (Table S3). Next, we constructed an overlap with the 1204 putative miR-433 target genes and identified four overlapping genes including CDK6, MAPK14, E2F3, and CDKN2A (Fig.2A). To further investigate the interaction between potential miR-433 targets and senescence-associated genes, we built an interaction network in Cytoscape using both ClueGo and Cluepedia apps. Only previously experimentally validated PPIs were included in the construction of the network. From this analysis, we identify a significant number of interactions between well-established cellular senescence genes and our list of miR-433 target genes (Fig.2B). By focusing in on the first degree neighbors of the four overlapping genes we identified a number of possible miR-433 targets which regulate the function of key cellular senescence genes such as Rb (Fig.2C). Overall, this indicated the possible role of miR-433 regulating the induction of cellular senescence in our stably transfected cells.

### Stable expression of miR-433 induces cellular senescence

**Figure 3:**
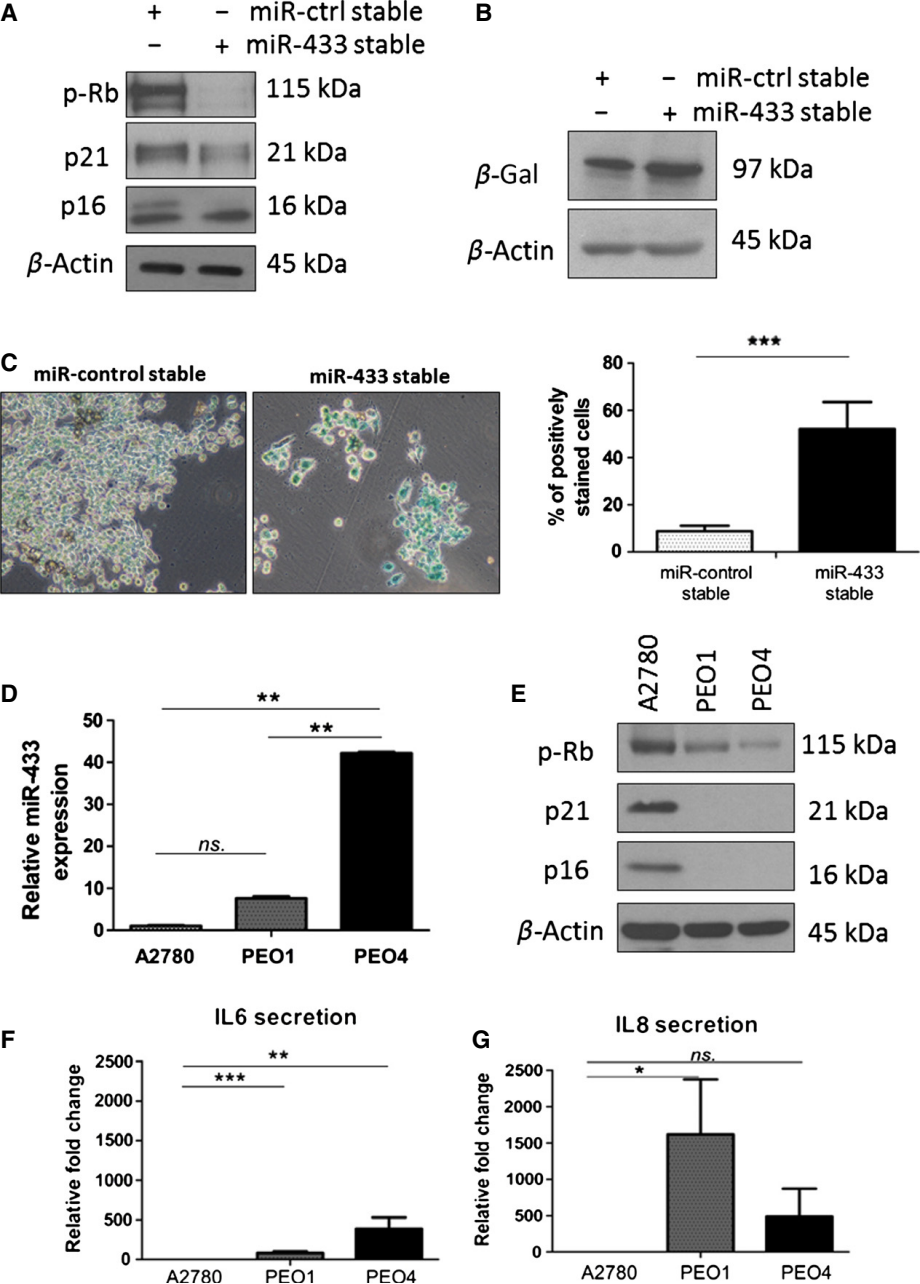
miR-433 stable expression induces cellular senescence. (A) Western blot analysis for p-Rb, p21, and p16 in miR-433-stable and miR-control-stable cell lines showing downregulation of p-RB with no demonstrable upregulation of p16 and p21. (B) Western blot analysis for *β*-galactosidase activity in miR-433-stable and miR-control-stable cell lines demonstrating an increase in *β*-galactosidase activity in miR-433 cells. (C) Senescence-dependent *β*-galactosidase activity staining in the miR-433-stable and miR-control-stable cell lines showing significant upregulation of positively stained cells in miR-433 cell line. (D) qRT-PCR determination of the baseline levels of miR-433 in the A2780, PEO1, and PEO4 ovarian cancer cell lines. (E) Western blot analysis of the baseline levels of expression of p-Rb, p21, and p16 in the A2780, PEO1, and PEO4 ovarian cancer cell lines showing a demonstrable decrease in p-Rb expression in the PEO1 and PEO4 paired cell line independent of p21 and p16 compared to A2780 cells. Error bars represent SEM. *P < 0.05, **P < 0.01, ***P < 0.001. (F and G) Analysis of cytokine secretion of interleukin-6 (IL-6), and interleukin-8 (IL-8) in the growth media of A2780, PEO1, and PEO4 cells, showing significant increases in IL-6 in PEO1 and PEO4 cells and IL-8 in PEO1 cells when compared to miR-433 negative A2780 cells. Error bars represent SEM. *P < 0.05, **P < 0.01, ***P < 0.001.

Following the in silico analysis of miR-433 targets, we investigated two key molecular pathways involved in the initiation and maintenance of cellular senescence namely, p53/p21 and p16/Rb 8. Firstly, to investigate if miR-433 overexpression could induce senescence the protein levels of p16, phosphorylated Rb (p-Rb), and p21 were anaylzed in the miR-433-stable A2780 cells. This analysis showed that p-Rb was decreased in A2780 cells stably transfected with miR-433. Notably, there was no reciprocal upregulation of p16 and p21 in these cells (Fig.3A). The senescence-associated *β*-galactosidase activity in these cells was determined by Western blot and *β*-galactosidase staining analyses and revealed a significant upregulation of senescence-associated *β*-galactosidase activity in miR-433-stable cells compared to controls (*P* < 0.0001) (Fig.3B and C).

To further explore the relationship between miR-433 expression and p-Rb, we profiled native miR-433 expression by qRT-PCR in the parent A2780 cells and two other epithelial ovarian cancer (EOC) lines, PEO1 and PEO4. PEO1 is cisplatin sensitive, while PEO4 is cisplatin resistant, derived from the same patient before and after the development of recurrent drug-resistant tumor 29. Here, we show that the cisplatin-resistant PEO4 cells displayed significantly higher levels of miR-433 compared to the A2780 (*P* = 0.0012) and PE01 cells (*P* = 0.0028) (Fig.3D). Additionally, when we analyzed the protein expression of p-Rb there was a demonstrable decrease in expression of p-Rb in the PEO1 and PEO4 compared to the A2780 cells with the cisplatin-resistant PEO4 line demonstrating the largest decrease in p-Rb expression across the three cell lines (Fig.3E). Of note, similar to our miR-433-stable cell line, the status of p-Rb in EOC cells was also independent of p21 or p16 expression (Fig.3A and E, respectively).

Senescent cells are known to contribute to the tumor microenvironment by secreting proinflammatory cytokines known as the senescence-associated secretory phenotype (SASP). To investigate this further, we measured the levels of interleukin-6 (IL-6) and interleukin-8 (IL-8) (two key cytokines associated with SASP) in the A2780, PEO1, and PEO4 cells using V-PLEX Human Proinflammatory (Meso Scale Discovery) kit (Fig.3F and G). Interestingly, the levels of IL-6 were significantly higher in the PEO1 and PEO4 cells compared to the A2780 cells (∼80-fold, *P* < 0.01 and ∼380-fold, *P* = 0.01, respectively) (Fig.3F) mirroring the increased levels of miR-433 in PEO1 and PEO4 compared to the A2780 cells (Fig.3D). Similarly, IL-8 levels were significantly higher in PEO1 cells compared to A2780 cells (∼1600-fold, *P* = 0.02) and although not reaching significance on IL-8 levels were on average ∼380-fold increased in PEO4 cells when compared with A2780 cells (*P* = 0.09) (Fig.3G).

### miR-433 regulates the expression of CDK6

**Figure 4:**
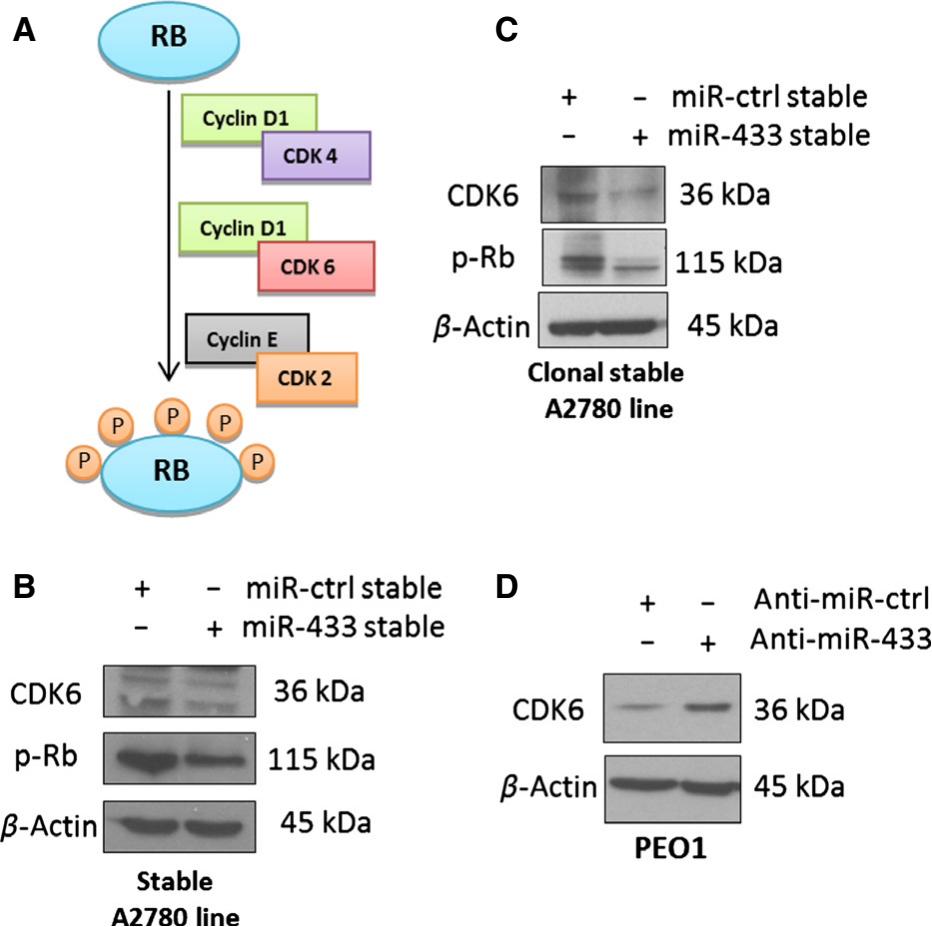
miR-433 induces senescence by targeting CDK6. (A) Schematic representation showing the published evidence of the phosphorylation of Rb by three independent cyclin-dependent kinase (CDK)/Cyclin complexes 30. (B) Western blot analysis for CDK6 expression in the miR-433-stable line demonstrating downregulation of CDK6. (C) Western blot analysis for CDK6 expression in the clonal derivative miR-433-stable line demonstrating downregulation of CDK6. (D) Western blot analysis for CDK6 reexpression in PEO1 cells transfected with anti-miR-control and anti-miR-433 for 96 h demonstrating an upregulation of CDK6.

As the data demonstrated that the miR-433-stable-cell-induced morphological changes (Fig.1E and F) with concomitant downregulation of p-Rb were *independent* of p16 and p21 (Fig.3A), we hypothesized that miR-433 may be directly targeting a kinase involved in the cell cycle-dependent phosphorylation of Rb. In this regard, it is known that phosphorylation of Rb in the G1 phase of the cell cycle is dependent on the activity of three complexes, namely, Cyclin D1/CDK4, Cyclin D1/CDK6, and Cyclin E/CDK2 (Fig.4A) 30. In our earlier bioinformatics analysis of potential miR-433 targets, CDK6 was predicted by five of the seven databases as a candidate miR-433 target gene. Therefore, we set out to establish if miR-433 could regulate the expression of CDK6. By analyzing protein expression in both the miR-433 stable A2780 cells and the clonal derivative of this cell line, we observed a decrease in CDK6 expression (Fig.4B and C, respectively). Additionally, transient overexpression of miR-433 in HeLa cells also demonstrated downregulation of CDK6 (Fig. S1). Moreover, the transient transfection of PEO1 cells with anti-miR-433 to inhibit miR-433, resulted in a demonstrable upregulation of CDK6 (Fig.4D). Overall these data suggest that miR-433-induced cellular senescence may be attributed to the loss of CDK6. Ultimately, this would result in cells having a reduced capacity to phosphorylate Rb, thereby, hindering progression through the cell cycle.

### High endogenous miR-433 expression attenuates apoptosis allowing cells to survive chemotherapy

**Figure 5:**
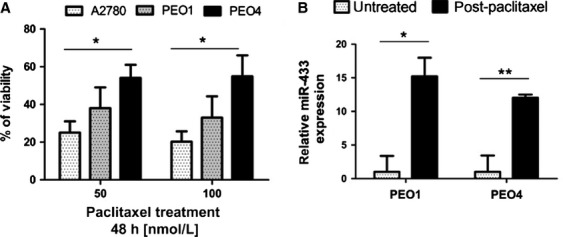
High miR-433 endogenous expression attenuates apoptosis with a resultant chemotherapy-induced senescence allowing cells to survive chemotherapy. (A) MTT viability assay demonstrating the % of growth inhibition of the A2780, PEO1, and PEO4 cells treated with paclitaxel (10, 25, and 50 nmol/L) for 48 h, demonstrating an increased resistance to paclitaxel treatment in PEO1 and PEO4 cells. (B) qRT-PCR analysis of miR-433 expression in PEO1 and PEO4 cells either untreated or treated with 50 nmol/L paclitaxel for 72 h, washed with PBS, and then cultured for 8 days in full growth medium showing an increased expression in cells treated with paclitaxel comparing to initial expression in untreated cells. Error bars represent SEM. *P < 0.05, **P < 0.01.

The relationship between endogenous miR-433 expression and chemoresistance to paclitaxel was investigated in the A2780, PEO1, and PEO4 cell lines where we demonstrated that chemosensitivity to paclitaxel correlated with miR-433 expression levels. Specifically, A2780 which has the lowest miR-433 expression (Fig.3D) is the most chemosensitive cell line in comparison to the more resistant PEO1 and PEO4 cells which have higher endogenous levels of miR-433 (Fig.5A). We then determined if cells that survive chemotherapy express increased levels of miR-433. PEO1 and PEO4 cells were treated with paclitaxel for 72 h after which fresh complete growth medium was added and the cells were cultured for a further 8 days. qRT-PCR analysis of the cells surviving chemotherapy demonstrated a significant upregulation of miR-433 expression in PEO1 by ∼15-fold (*P* = 0.024) and in PEO4 by ∼12-fold (*P* = 0.0053) compared to untreated cells (Fig.5B). However,we did not observe statistical difference in the miR-433 expression after short 24 and 48 h paclitaxel treatment (50 nmol/L) in both PEO1 (*P* = 0.155 and *P* = 0.55) and PEO4 cells (*P* = 0.24 and *P* = 0.36) (Fig. S2). These data suggest that miR-433 is not acutely upregulated by paclitaxel treatment. However, significant upregulation of miR-433 8 days post paclitaxel treatment suggests that only cells with the highest miR-433 expression survive chemotherapy. Interestingly, PEO4 cells surviving paclitaxel treatment also demonstrated senescence evidenced by increased *β*-galactosidase activity (Fig. S2B). In the clinical setup, one could envisage that these miR-433 enriched cells through environmental selection over time, constitute chemoresistant clones capable of seeding recurrent disease.

### miR-433 expression impacts on cellular senescence induction in the tumor microenvironment

**Figure 6:**
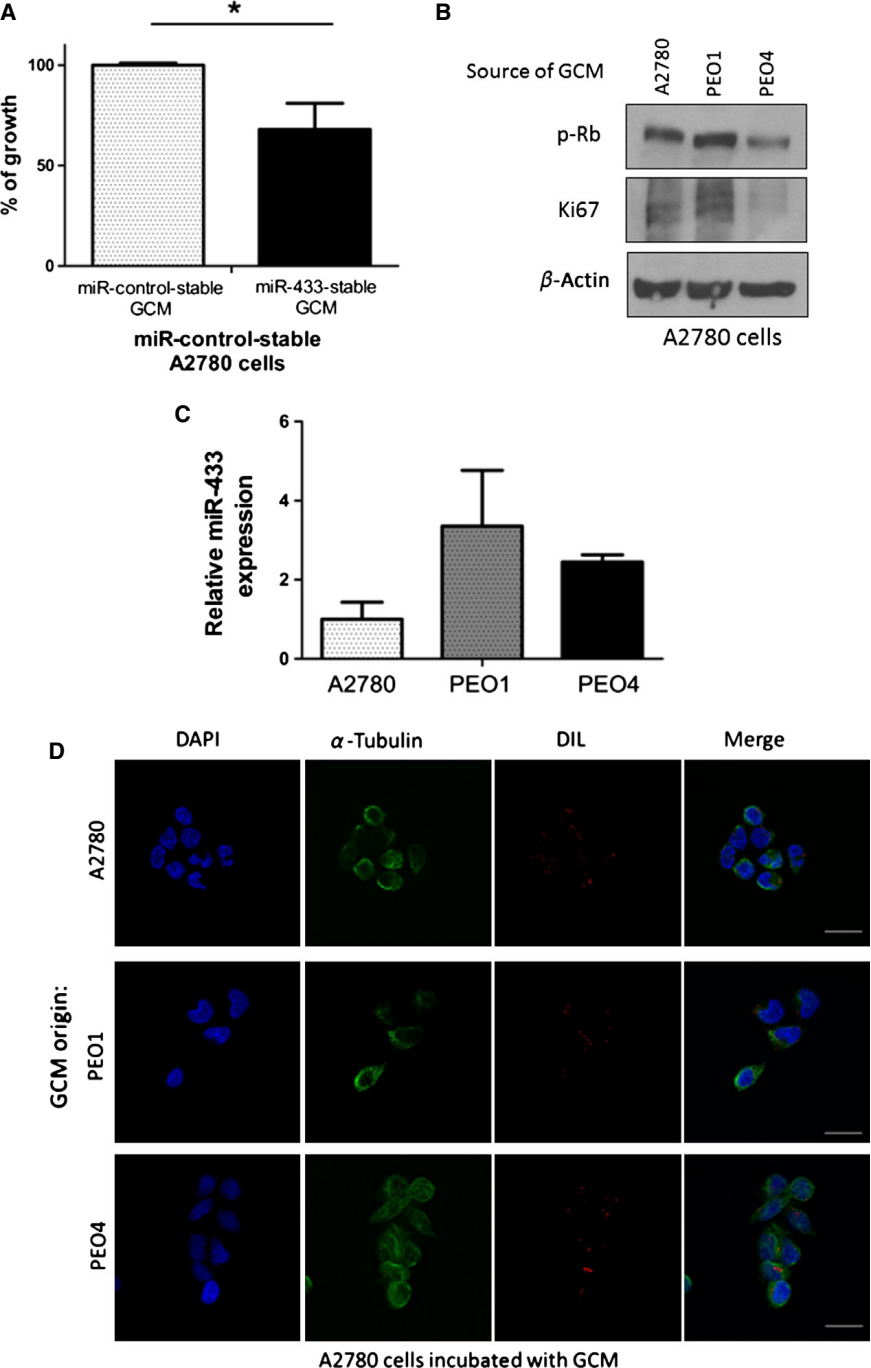
miR-433 expression has the potential to induce cellular senescence in the tumor microenvironment. (A) MTT viability assay demonstrating a decreased viability in miR-control-stable cells treated with growth conditioned medium (GCM) from stable miR-433 expressing cells compared to being cultured in control medium. (B) Western blot analysis of the expression of senescent markers of A2780 cells treated with GCM from A2780, PEO1, or PEO4 cells demonstrating a marked reduction in p-RB and Ki67 when A2780 cells are grown in conditioned medium from PEO4 cell line known to express the highest baseline levels of miR-433 (Fig.3C). (C) Relative miR-433 expression in exosomes derived from GCM harvested from A2780, PEO1, and PEO4 cells was assessed by TaqMan® qRT-PCR using the comparative C_T_ (ΔΔC_T_) method. miR-433 expression was present in the exosomes derived from all cell lines. (D) Fluorescent micrograph showing a successful incorporation of Dil-labeled vesicles from A2780, PEO1, or PEO4 GCM (shown in red) inside the recipient A2780 cells, counterstained with DAPI nuclear staining (shown in blue) and microtubules with Alexa Fluor 488 *α*-tubulin staining (shown in green). Error bars represent SEM. *P < 0.05.

Senescent cells are known to directly impact on the tumor microenvironment 31. Therefore, we set out to determine what effect GCM harvested from the miR-433-stable transfected cells would have when used to culture the parent A2780 cells. Results demonstrated that A2780 cells cultured in the GCM from miR-433 stable expressing cells had a significantly increased growth inhibition (MTT) compared to control cells (*P* = 0.012) (Fig.6A). Similarly, GCM from both PEO1 and PEO4 cells, which display increased levels of miR-433, also influenced the growth of control parent A2780 cells (Fig.6B). Interestingly, we observed a decrease in the expression levels of p-Rb and the proliferation marker Ki67 in A2780 cells incubated with GCM from PEO4 cells, the cisplatin-resistant derivative of PEO1 and the cell line known to have the highest levels of miR-433 (Fig.6B).

It is well established that paracrine signaling between senescent cells and the tumor microenvironment can occur through the release of soluble factors such as cytokines or through the release of microvesicles or exosomes in various cancer cell lines including lung, prostate, breast, and erythroleukaemic 32–35. Therefore, we investigated if miR-433 could be present in exosomes. Exosomes were isolated from the miR-433-stable and miR-control A2780 cells. Successful exosome isolation was confirmed through nanoparticle tracking technology (NanoSight®) and the expression of miR-433 quantified using qRT-PCR (Fig. S3A–C). From this we showed that cells with stable expression of miR-433 also have increased miR-433 levels in the exosomes which they release (Fig. S3D). Simillarily, A2780, PEO1, and PEO4 exosomes were isolated from GCM and confirmed through nanoparticle tracking technology (NanoSight®) (Fig. S3E–G) and the expression of miR-433 quantified with qRT-PCR. Interestingly, we observed miR-433 expression in the exosomes derived from each cell line (Fig.6C).

To investigate if miR-433 could potentially be transported by vesicles, GCM from the A2780, PEO1, and PEO4 cells were centrifuged to remove any dead cells and cellular debris and subsequently labeled with the lipophilic dye Dil. Using confocal microscopy, vesicle uptake was observed in parent A2780 cells cultured in the presence of labeled GCM from all three cell lines (Fig.6C). In summary, these data indicate that miR-433-enriched cells have the ability to affect their tumor microenvironment and to induce cellular senescence in neighboring cells potentially by miR-433 incorporated into vesicles.

## Discussion

The contribution of altered miRNA expression to a pathology such as cancer is now widely accepted. However, deciphering the exact function of a miRNA has proven more challenging. We present data demonstrating a functional role of miR-433 in the induction of cellular senescence, thereby conferring resistance to paclitaxel in ovarian cancer cells. Our results indicated that miR-433 expression leads to functional inactivation of the Rb protein, followed by the disruption of cell cycle progression and the induction of cellular senescence. The induction of cellular senescence by miR-433 may initially act as a protective mechanism and thus arrest actively dividing cells 8. Indeed, in gastric cancer cells, miR-433 has been attributed with tumor suppressor functions 20. However, our data indicate that once a cancer cell has committed to a senescent phenotype, miR-433 can mediate resistance to paclitaxel 5. Of note is our finding that cells surviving chemotherapy treatment are enriched in miR-433. As paclitaxel treatment did not induce miR-433 expression, we suggest that miR-433 enrichment in post chemotherapy treated cell populations, is associated with the survival of cells with the highest endogenous miR-433 and resultant senescent phenotype.

Clinically, cellular senescence-associated with the expression of the p16/p-Rb pathway is widely present in HGSOC patients where a meta-analysis of multivariate estimates revealed a significant association between high p16 expression and poor PFS 36. The TCGA project has also identified the Rb pathway as a major contributor to HGSOC pathogenesis 37. Recently, it has been shown that functionally inactive Rb has a fundamental role in promoting chromosome instability (CIN) 38. CIN coupled with its consequential chromosomal aneuploidy is a main feature of advanced tumor grade and is strongly associated with poor response to chemotherapies and further tumor evolution 39. Manning et al.*,* have shown that the induction of CIN is dependent on the synergistic inactivation/mutation of both Rb and p53 38. Strikingly, 95% of all ovarian tumors have p53 mutations 37. Therefore, miR-433-dependent functional silencing of Rb (or in other words downregulation of p-Rb) in p53-deregulated ovarian tumors could promote CIN and contribute to further tumor development. Importantly, our group has previously published that downregulation of the miR-433 target, MAD2 and promoted anaphase bridges formation which is a prerequisite to chromosomal aneuploidy 21. Consequently, the synergistic effect of miR-433-dependent inactivation of Rb (possibly through CDK6) and MAD2 may contribute to CIN in HGSOC.

Our bioinformatics analysis showed that miR-433 has significant potential to regulate senescence-associated proteins. Importantly, downregulation of four of these genes namely: hPOT1, CREB1, Aurora A, and TOP1 has already been shown to induce cellular senescence 40–43. Downregulation of others such as RAD21, TRIP12, E2F3, MED1, SORBS2, HIPK2, SMC1A, and IQGAP1 has also been associated with cell cycle inhibition. Importantly, in relation to miR-433, two independent studies have demonstrated that indeed, miR-433 expression is associated with migration and invasion inhibition 17,20. Additionally, Guo et al. associated miR-433 expression levels with a significant inhibition of cell cycle progression in HGC-27 gastric cancer cells 20. In our study, for the first time we demonstrate that stable miR-433 expression is also associated with the induction of cellular senescence in A2780 ovarian cancer cells. Furthermore, we show that senescence induction in our model resulted from the downregulation of CDK6 in a p21 and p16 independent manner. Senescence activation, independent from canonical pathways (p53/p21 and p16/Rb) has been reported previously 44,45. Specifically, Rader et al. demonstrated that concomitant downregulation of both CDK4/6 by either siRNA or the pharmacological kinase inhibitor LEE011 caused cell cycle arrest, followed by senescence in neuroblastoma 44. Conversely, Anders et al. have demonstrated that CDK4/6-dependent activation of the FOXM1 transcription factor suppressed senescence by an activation of critical G1/S genes promoting S phase entry in melanoma cells 45. Taking these data together suggests that functional inactivation of CDK4/6 is an important mechanism for cellular senescence induction in various cancers.

Our data also indicate that miR-433 expressing cells have the ability to affect their tumor microenvironment by diminishing the proliferation in neighboring cells with the induction of cellular senescence. The induction of cellular senescence and subsequent altered cell signaling has been shown to correlate with changes in the epigenome of cells and to promote further cancer progression 46. Yang and colleagues demonstrated that coinjection of senescent ovarian fibroblasts with premalignant epithelial cells into mice induced progression of senescence-dependent carcinogenesis in vivo 47. Additionally, senescent cells secrete large amounts of signaling molecules, a feature known as the SASP 7. In particular, IL6 and IL8 are known to correlate with poor survival and further cancer development in patients presenting with EOC 48. Therefore, the induction of senescence by miR-433 may act to reinforce the cell's response to uncontrollable cell proliferation but ultimately the resultant senescent phenotype may adversely affect the progression of the cancer and patient survival.

In summary, this study has demonstrated how dysregulation of a single microRNA, miR-433, through its regulation of multiple protein targets results in the modulation of cell signaling pathways to induce cellular senescence and resistance to paclitaxel.

## Supporting Information

**Figure S1.** (A) Western blot analysis of CDK6 expression following transient transfection of pre-miR-433 (100 nmol/L) for 24 h in HeLa cells demonstrating the downregulation of CDK6 in cells transfected with pre-miR-433. (B) Fluorescent micrograph showing a successful incorporation of a Cy3-labeled anti-miR-control (shown in red) inside the cells with DAPI nuclear staining shown in blue. Cells were seeded into the four-well chamber slides and transfected with 100 nmol/L of Cy3-labeled anti-miR-control for 48 h. Subsequently the cells were fixed counterstained with DAPI and visualized using 405 nm excitation beam for DAPI and 559 nm for Cy3.

**Figure S2.** (A) qRT-RCR analysis of miR-433 expression in A2780, PEO1, and PEO4 cells treated with 50 nmol/L paclitaxel for 24 and 48 h. (B) Senescence-dependent *β*-galactosidase activity staining on the PEO1 and PEO4 cells treated with paclitaxel for 72 h and then recovered in full growth medium for 8 days demonstrating and increase in positively stained PEO4 cells.

**Figure S3.** miR-433 is present in exosomes harvested from miR-433-stable GCM. (A) Size distribution of vesicles isolated from miR-control and miR-433 stable cells analyzed by Nanoparticle Tracking Technology—NanoSight® demonstrating the successful isolation of vesicles ranging from 30 to 400 nm in both cell lines. (B) Graph representing no significant difference between mean and mode size distribution of the vesicles derived from GCM of either miR-control and miR-433 stable cell quantified by Nanoparticle Tracking Technology—NanoSight® (C) Quantification of exosomes derived from GCM of either miR-control or miR-433 stable cells calculated with Nanoparticle Tracking Technology—NanoSight® demonstrating no significant difference in the amount of exosomes released per cells between these cell lines. (D) Relative miR-433 expression in exosomes derived from GCM harvested from miR-control or miR-433 stable cells was assessed by TaqMan® qRT-PCR using the comparative C_T_ (ΔΔC_T_) method. Increased miR-433 expression was observed in the A2780 stably expressing miR-433 cell line compared to scrambled control. Increased miR-433 expression was only present in the miR-433 stable cells and exosomes derived from these miR-433 stable cells. (E) Size distribution of vesicles isolated from PEO1 and PEO4 cells analyzed by Nanoparticle Tracking Technology—NanoSight® demonstrating the successful isolation of vesicles ranging from 30 to 400 nm in both cell lines. (F) Graph representing no significant difference between mean and mode size distribution of the vesicles derived from GCM of either PEO1 and PEO4 stable cell quantified by Nanoparticle Tracking Technology—NanoSight® (G) Quantification of exosomes derived from GCM of either PEO1 or PEO4 cells calculated with Nanoparticle Tracking Technology—NanoSight® demonstrating no significant difference in the amount of exosomes released per cells between these cell lines. Error bars represent SEM. **P* < 0.05, ***P* < 0.01, ****P* < 0.001.

**Table S1.** Identification of candidate target genes of miR-433 using seven different prediction databases. Genes positively identified by five of seven databases were used for further analysis. **Table S2.** KEGG pathway analysis of high confidence miR-433 predicted target genes. **Table S3.** List of genes which have previously been associated with cellular senescence.
