# Supplementary figures and images for: Overexpression of the microRNA miR-433 promotes resistance to paclitaxel through the induction of cellular senescence in ovarian cancer cells
Source: Cancer Med. 2015 Feb 15;4(5):745–58. doi: 10.1002/cam4.409 (PMC4430267; doi:10.1002/cam4.409)

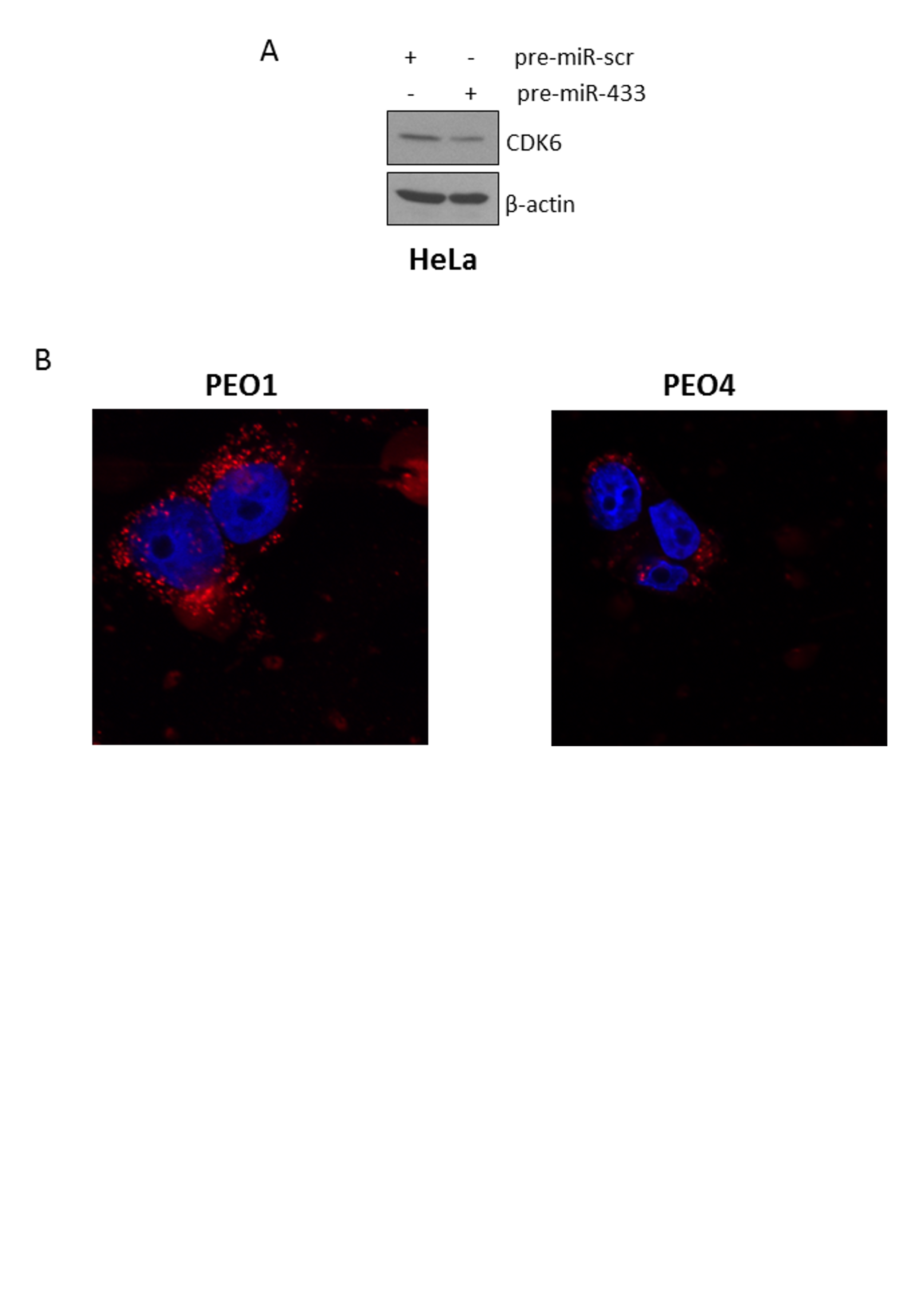

Supplement: Supplementary file 1 — Figure S1. (A) Western blot analysis of CDK6 expression following transient transfection of pre-miR-433 (100 nmol/L) for 24 h in HeLa cells demonstrating the downregulation of CDK6 in cells transfected with pre-miR-433. (B) Fluorescent micrograph showing a successful incorporation of a Cy3-labeled anti-miR-control (shown in red) inside the cells with DAPI nuclear staining shown in blue. Cells were seeded into the four-well chamber slides and transfected with 100 nmol/L of Cy3-labeled anti-miR-control for 48 h. Subsequently the cells were fixed counterstained with DAPI and visualized using 405 nm excitation beam for DAPI and 559 nm for Cy3. [file cam40004-0745-sd1.tif]

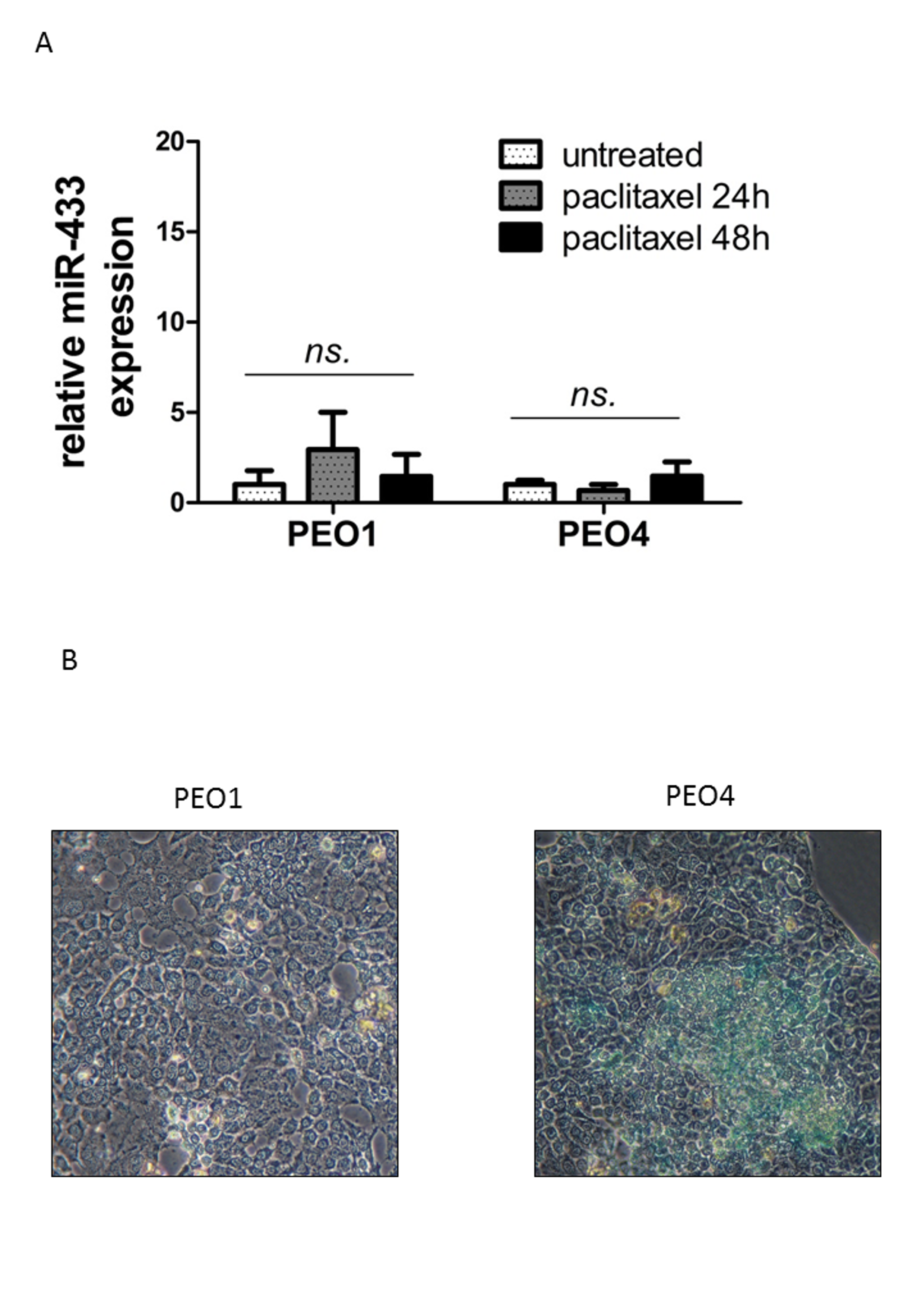

Supplement: Supplementary file 2 — Figure S2. (A) qRT-RCR analysis of miR-433 expression in A2780, PEO1, and PEO4 cells treated with 50 nmol/L paclitaxel for 24 and 48 h. (B) Senescence-dependent β-galactosidase activity staining on the PEO1 and PEO4 cells treated with paclitaxel for 72 h and then recovered in full growth medium for 8 days demonstrating and increase in positively stained PEO4 cells. [file cam40004-0745-sd2.tif]

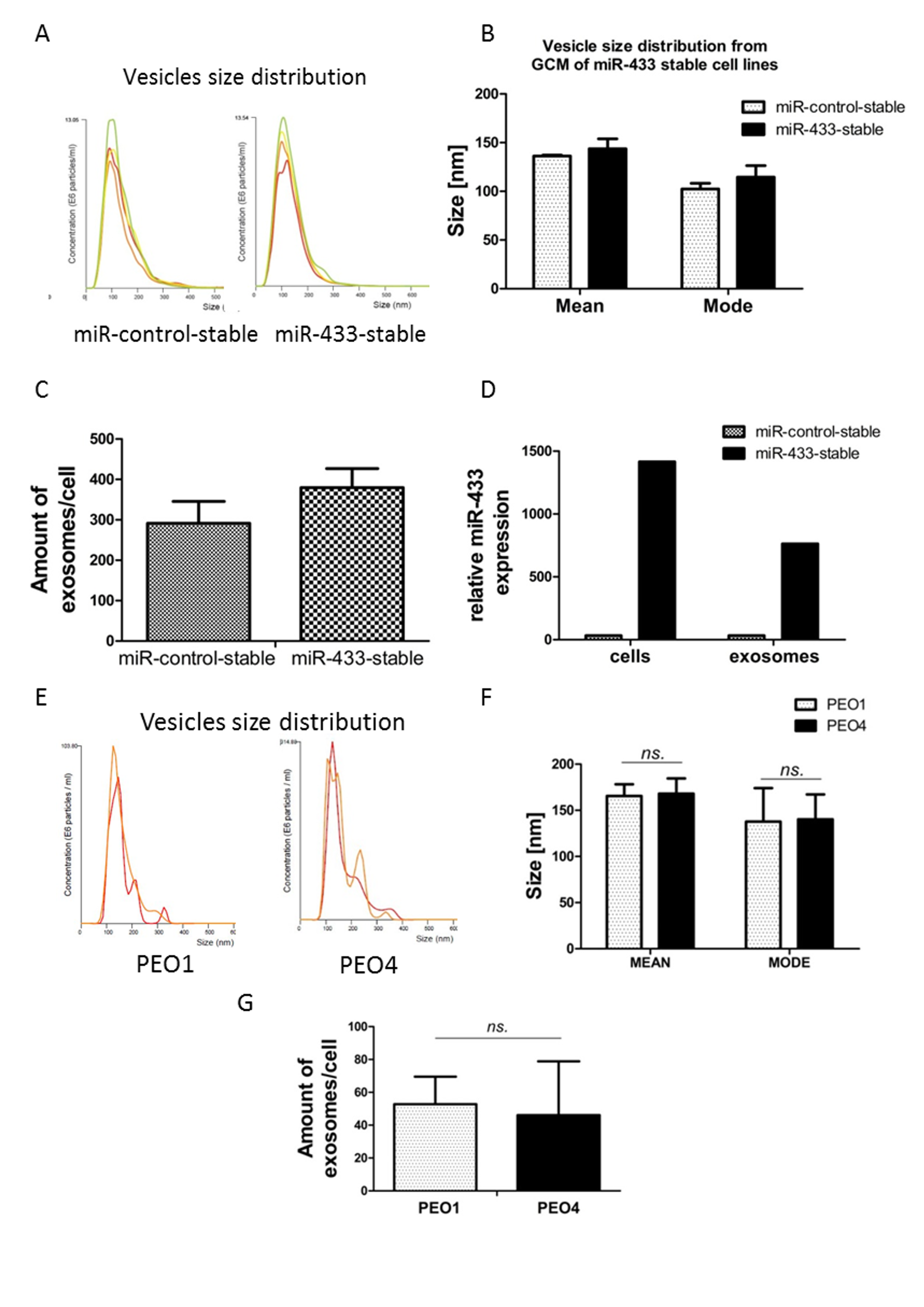

Supplement: Supplementary file 3 — Figure S3. miR-433 is present in exosomes harvested from miR-433-stable GCM. (A) Size distribution of vesicles isolated from miR-control and miR-433 stable cells analyzed by Nanoparticle Tracking Technology—NanoSight® demonstrating the successful isolation of vesicles ranging from 30 to 400 nm in both cell lines. (B) Graph representing no significant difference between mean and mode size distribution of the vesicles derived from GCM of either miR-control and miR-433 stable cell quantified by Nanoparticle Tracking Technology—NanoSight® (C) Quantification of exosomes derived from GCM of either miR-control or miR-433 stable cells calculated with Nanoparticle Tracking Technology—NanoSight® demonstrating no significant difference in the amount of exosomes released per cells between these cell lines. (D) Relative miR-433 expression in exosomes derived from GCM harvested from miR-control or miR-433 stable cells was assessed by TaqMan® qRT-PCR using the comparative CT (ΔΔCT) method. Increased miR-433 expression was observed in the A2780 stably expressing miR-433 cell line compared to scrambled control. Increased miR-433 expression was only present in the miR-433 stable cells and exosomes derived from these miR-433 stable cells. (E) Size distribution of vesicles isolated from PEO1 and PEO4 cells analyzed by Nanoparticle Tracking Technology—NanoSight® demonstrating the successful isolation of vesicles ranging from 30 to 400 nm in both cell lines. (F) Graph representing no significant difference between mean and mode size distribution of the vesicles derived from GCM of either PEO1 and PEO4 stable cell quantified by Nanoparticle Tracking Technology—NanoSight® (G) Quantification of exosomes derived from GCM of either PEO1 or PEO4 cells calculated with Nanoparticle Tracking Technology—NanoSight® demonstrating no significant difference in the amount of exosomes released per cells between these cell lines. Error bars represent SEM. *P < 0.05, **P < 0.01, ***P < 0.001. [file cam40004-0745-sd3.tif]
